# Supplementary material for: Effect of Botulinum Toxin Injections in the Treatment of Spasticity of Different Etiologies: An Umbrella Review
Source: Pharmaceuticals (Basel). 2024 Feb 28;17(3):310. doi: 10.3390/ph17030310 (PMC10976034; doi:10.3390/ph17030310)
Supplement: Supplementary file 1 [file pharmaceuticals-17-00310-s001.zip › pharmaceuticals-2882773-supplementary.pdf]

SUPPLEMENTARY MATERIAL

Supplementary Table 1: Modified Ashworth Scale.

0	No increase muscle tone
1	Slight increase in muscle tone, with a catch and release or minimal resistance at the end of the range of motion when an affected part(s) is moved in flexion or extension
1+	Slight increase in muscle tone, manifested as a catch, followed by minimal resistance through the remainder (less than half) of the range of motion
2	More marked increase in muscle tone throughout most of the range of motion, but affected part(s) are still easily moved
3	Considerable increase in muscle tone, passive movement difficult
4	Affected part(s) rigid in flexion or extension

Adapted from Yam W,K and Leung. [5]

Supplementary Table 2:

**Quality of muscle reaction**	
0	No resistance throughout the course of the passive movement
1	Slight resistance through the course of passive movement: no clear "catch" at a precise angle
2	Clear catch at a precise angle, interrupting the passive movement, followed by release
3	Fatiguable clonus (< 10s when maintaining the pressure) appearing at a precise angle
4	Unfatiguable clonus (>10s when maintaining the pressure) at a precise angle
5	Joint immovable
**Joint angles**	
Measure relative to the position of minimal stretch of the muscle (corresponding to angle zero) for all joints except the hip, where it is relative to the resting anatomic position (eg, angle zero corresponds to the ankle at 90 deg and the hip at midline)	
R1	Angle of muscle reaction
R2	Angle of full range of motion (passive range of motion)
**Definition of velocities used**	
V1	As slow as possible (slower than the natural drop of the limb segment under gravity)
V2	Speed of the limb segment falling under gravity
V3	As fast as possible (faster than the rate the natural drop of the limb segment under gravity)

Adapted from Yam W,K and Leung. [5]

Supplementary Table 3: Search strategy

Children<br>**OR**<br>Adults	**AND**	Botulinum toxin<br>**OR**<br>Botox A	**AND**	Cerebral palsy<br>**OR**<br>Spasticity<br>**OR**<br>Spastic paraplegia	**AND**	Systematic Review<br>**OR**<br>RS<br>**OR**<br>Meta-analysis<br>**OR**<br>Network meta-analysis

Supplementary Table 4: Assessment of the methodological quality of the included systematic reviews using the AMSTAR 2 tool.

**Reference**	**1**	**2**	**3**	**4**	**5**	**6**	**7**	**8**	**9**	**10**	**11**	**12**	**13**	**14**	**15**	**16**	**Risk of bias**
Reeuwijk A et al (2006)	Y	PY	Y	Y	Y	Y	Y	Y	Y	N	N	NA	Y	Y	NA	N	Critically low
Demetrios M et al (2013)	Y	Y	Y	Y	Y	Y	Y	Y	Y	N	NA	NA	Y	Y	NA	Y	Moderate
Phadke CP et al (2014)	Y	PY	Y	Y	Y	Y	Y	Y	NA	Y	NA	NA	Y	Y	NA	Y	Moderate
García Salazar LF et al (2015)	Y	PY	Y	Y	Y	Y	Y	Y	Y	N	NA	NA	Y	N	NA	Y	Moderate
Dashtipour K et al (2015)	Y	Y	Y	Y	N	N	Y	Y	Y	Y	NA	NA	Y	Y	NA	Y	Moderate
Fonseca Junior PR et al (2017)	Y	PY Y	Y	Y	Y	Y	Y	Y	Y	N	NA	NA	Y	Y	NA	Y	Moderate
Gupta AD et al (2018)	Y	Y	Y	Y	Y	Y	Y	Y	Y	N	NA	NA	Y	Y	NA	Y	Moderate

Yana M et al (2019)	Y	PY	Y	Y	Y	Y	Y	Y	Y	N	NA	NA	Y	Y	NA	Y	Moderate
Hara T et al (2019)	Y	PY	Y	Y	Y	Y	Y	Y	Y	N	NA	NA	Y	Y	NA	Y	Moderate
Farag SM et al (2020)	Y	PY	Y	Y	Y	Y	Y	Y	Y	Y	NA	NA	Y	N	NA	Y	Moderate
Klein C et al (2023)	Y	PY	Y	Y	Y	Y	Y	Y	Y	Y	NA	NA	Y	N	NA	Y	Moderate
Yang H et al (2023)	Y	Y	Y	Y	Y	Y	Y	Y	Y	N	NA	NA	Y	Y	NA	Y	Moderate

**Reference**	**1**	**2**	**3**	**4**	**5**	**6**	**7**	**8**	**9**	**10**	**11**	**12**	**13**	**14**	**15**	**16**	**Risk of bias**
Reeuwijk A et al (2006)	Y	PY	Y	Y	Y	Y	Y	Y	Y	N	N	CA	Y	Y	CA	N	Critically low
Demetrios M et al (2013)	Y	Y	Y	Y	Y	Y	Y	Y	Y	N	CA	CA	Y	Y	CA	Y	Moderate
Phadke CP et al (2014)	Y	PY	Y	Y	Y	Y	Y	Y	CA	Y	CA	CA	Y	Y	CA	Y	Moderate
García Salazar LF et al (2015)	Y	PY	Y	Y	Y	Y	Y	Y	Y	N	CA	CA	Y	N	CA	Y	Moderate
Dashtipour K et al (2015)	Y	Y	Y	Y	N	N	Y	Y	Y	Y	CA	CA	Y	Y	CA	Y	Moderate
Fonseca Junior PR et al (2017)	Y	PY Y	Y	Y	Y	Y	Y	Y	Y	N	CA	CA	Y	Y	CA	Y	Moderate
Gupta AD et al (2018)	Y	Y	Y	Y	Y	Y	Y	Y	Y	N	CA	CA	Y	Y	CA	Y	Moderate
Yana M et al (2019)	Y	PY	Y	Y	Y	Y	Y	Y	Y	N	CA	CA	Y	Y	CA	Y	Moderate
Hara T et al (2019)	Y	PY	Y	Y	Y	Y	Y	Y	Y	N	CA	CA	Y	Y	CA	Y	Moderate
Farag SM et al (2020)	Y	PY	Y	Y	Y	Y	Y	Y	Y	Y	CA	CA	Y	N	CA	Y	Moderate

Klein C et al (2023)	Y	PY	Y	Y	Y	Y	Y	Y	Y	Y	CA	CA	Y	N	CA	Y	Moderate
Yang H et al (2023)	Y	Y	Y	Y	Y	Y	Y	Y	Y	N	CA	CA	Y	Y	CA	Y	Moderate

Y: yes; N: no; CA: cannot answer; PY: partial yes. 1) Did the research questions and inclusion criteria for the review include the components of PICO? 2) Did the report of the review contain an explicit statement that the review methods were established prior to the conduct of the review and did the report justify any significant deviations from the protocol? 3) Did the review authors explain their selection of the study designs for inclusion in the review? 4) Did the review authors use a comprehensive literature search strategy? 5) Did the review authors perform study selection in duplicate? 6) Did the review authors perform data extraction in duplicate? 7) Did the review authors provide a list of excluded studies and justify the exclusions? 8) Did the review authors describe the included studies in adequate detail? 9) Did the review authors use a satisfactory technique for assessing the risk of bias (RoB) in individual studies that were included in the review? 10) Did the review authors report on the sources of funding for the studies included in the review? 11) If meta-analysis was performed did the review authors use appropriate methods for statistical combination of results? 12) If meta-analysis was performed, did the review authors assess the potential impact of RoB in individual studies on the results of the meta-analysis or other evidence synthesis? 13) Did the review authors account for RoB in individual studies when interpreting/ discussing the results of the review? 14) Did the review authors provide a satisfactory explanation for, and discussion of, any heterogeneity observed in the results of the review? 15) If they performed quantitative synthesis did the review authors carry out an adequate investigation of publication bias (small study bias) and discuss its likely impact on the results of the review? 16) Did the review authors report any potential sources of conflict of interest, including any funding they received for conducting the review?

Supplementary Table 5: Assessment of the methodological quality of the included meta-analyses using the AMSTAR 2 tool.

**Reference**	**1**	**2**	**3**	**4**	**5**	**6**	**7**	**8**	**9**	**10**	**11**	**12**	**13**	**14**	**15**	**16**	**Risk of bias**
Boyd R and Hays R (2001)	Y	Y	Y	Y	N	N	Y	Y	N	N	Y	N	N	Y	N	N	Critically Low
Wasiak J et al (2004)	Y	Y	Y	Y	Y	Y	Y	Y	PY	N	Y	N	N	Y	Y	N	Low
Cardoso E et al (2005)	Y	Y	Y	Y	Y	Y	Y	Y	Y	N	Y	Y	N	Y	Y	N	Low
Rosales R et al (2008)	Y	Y	Y	Y	Y	Y	Y	Y	Y	N	Y	Y	Y	Y	Y	N	Moderate
Elia AE et al (2009)	Y	Y	Y	Y	Y	Y	Y	Y	Y	Y	N	Y	Y	Y	Y	Y	High
Koog YH and Min BI (2010)	Y	Y	Y	Y	Y	Y	Y	Y	Y	Y	Y	Y	Y	Y	Y	N	High

Baker JA and Pereira G (2013)	Y	Y	Y	Y	N	N	Y	Y	Y	Y	Y	Y	Y	Y	Y	Y	Moderate
Wu T et al (2016)	Y	Y	Y	Y	Y	Y	Y	Y	Y	N	Y	Y	Y	Y	Y	Y	High
Dong Y et al (2017)	Y	Y	Y	Y	Y	Y	Y	Y	Y	N	Y	Y	Y	Y	Y	Y	High
Guyot P et al (2019)	Y	Y	Y	Y	Y	Y	Y	Y	Y	N	Y	Y	Y	Y	Y	Y	High
Blumetti FC et al (2019)	Y	Y	Y	Y	Y	Y	Y	Y	Y	Y	Y	Y	Y	Y	Y	Y	High
Sun LC et al (2019)	Y	Y	Y	Y	Y	Y	Y	Y	Y	N	Y	Y	Y	Y	Y	Y	High
Jia S et al (2020)	Y	Y	Y	Y	Y	Y	Y	Y	Y	N	Y	Y	Y	Y	Y	Y	High
Doan TN et al (2021)	Y	Y	Y	Y	Y	Y	Y	Y	Y	N	Y	Y	Y	Y	Y	Y	High
Varvarousis DN et al (2021)	Y	Y	Y	Y	Y	Y	Y	Y	Y	N	Y	Y	Y	Y	Y	Y	High
Ojardias E et al (2022)	Y	Y	Y	Y	Y	Y	Y	Y	Y	N	Y	Y	Y	Y	Y	Y	High

**Reference**	**1**	**2**	**3**	**4**	**5**	**6**	**7**	**8**	**9**	**10**	**11**	**12**	**13**	**14**	**15**	**16**	**Risk of bias**
Boyd R and Hays R (2001)	Y	Y	Y	Y	N	N	Y	Y	N	N	Y	N	N	Y	N	N	Critically Low
Wasiak J et al (2004)	Y	Y	Y	Y	Y	Y	Y	Y	PY	N	Y	N	N	Y	Y	N	Low
Cardoso E et al (2005)	Y	Y	Y	Y	Y	Y	Y	Y	Y	N	Y	Y	N	Y	Y	N	Low

Rosales R et al (2008)	Y	Y	Y	Y	Y	Y	Y	Y	Y	N	Y	Y	Y	Y	Y	N	Moderate
Elia AE et al (2009)	Y	Y	Y	Y	Y	Y	Y	Y	Y	Y	N	Y	Y	Y	Y	Y	High
Koog YH and Min BI (2010)	Y	Y	Y	Y	Y	Y	Y	Y	Y	Y	Y	Y	Y	Y	Y	N	High
Baker JA and Pereira G (2013)	Y	Y	Y	Y	N	N	Y	Y	Y	Y	Y	Y	Y	Y	Y	Y	Moderate
Wu T et al (2016)	Y	Y	Y	Y	Y	Y	Y	Y	Y	N	Y	Y	Y	Y	Y	Y	High
Dong Y et al (2017)	Y	Y	Y	Y	Y	Y	Y	Y	Y	N	Y	Y	Y	Y	Y	Y	High
Guyot P et al (2019)	Y	Y	Y	Y	Y	Y	Y	Y	Y	N	Y	Y	Y	Y	Y	Y	High
Blumetti FC et al (2019)	Y	Y	Y	Y	Y	Y	Y	Y	Y	Y	Y	Y	Y	Y	Y	Y	High
Sun LC et al (2019)	Y	Y	Y	Y	Y	Y	Y	Y	Y	N	Y	Y	Y	Y	Y	Y	High
Jia S et al (2020)	Y	Y	Y	Y	Y	Y	Y	Y	Y	N	Y	Y	Y	Y	Y	Y	High
Doan TN et al (2021)	Y	Y	Y	Y	Y	Y	Y	Y	Y	N	Y	Y	Y	Y	Y	Y	High
Varvarousis DN et al (2021)	Y	Y	Y	Y	Y	Y	Y	Y	Y	N	Y	Y	Y	Y	Y	Y	High
Ojardias E et al (2022)	Y	Y	Y	Y	Y	Y	Y	Y	Y	N	Y	Y	Y	Y	Y	Y	High

Y: yes; N: no; CA: cannot answer; PY: partial yes. 1) Did the research questions and inclusion criteria for the review include the components of PICO? 2) Did the report of the review contain an explicit statement that the review methods were established prior to the conduct of the review and did the report justify any significant deviations from the protocol? 3) Did the review authors explain their selection of the study designs for inclusion in the review? 4) Did the review authors use a comprehensive literature search strategy? 5) Did the review authors perform study selection in duplicate? 6) Did the review authors perform data extraction in

duplicate? 7) Did the review authors provide a list of excluded studies and justify the exclusions? 8) Did the review authors describe the included studies in adequate detail? 9) Did the review authors use a satisfactory technique for assessing the risk of bias (RoB) in individual studies that were included in the review? 10) Did the review authors report on the sources of funding for the studies included in the review? 11) If meta-analysis was performed did the review authors use appropriate methods for statistical combination of results? 12) If meta-analysis was performed, did the review authors assess the potential impact of RoB in individual studies on the results of the meta-analysis or other evidence synthesis? 13) Did the review authors account for RoB in individual studies when interpreting/ discussing the results of the review? 14) Did the review authors provide a satisfactory explanation for, and discussion of, any heterogeneity observed in the results of the review? 15) If they performed quantitative synthesis did the review authors carry out an adequate investigation of publication bias (small study bias) and discuss its likely impact on the results of the review? 16) Did the review authors report any potential sources of conflict of interest, including any funding they received for conducting the review?

Supplementary table 6: Quality grading of evidence.

Certainty assessment							№ of patients		Effect		Certainty	Importance
**№ of studies**	**Study design**	**Risk of bias**	**Inconsistency**	**Indirectness**	**Imprecision**	**Other considerations**	**[Intervention]**	**[Comparator]**	**Relative (95% CI)**	**Absolute (95% CI)**		
Waisak J et al (2004) [40]												
**10**	randomised trials	very serious[a]	very serious[a]	not serious	not serious	publication bias strongly suspected[a]	15	14	-	SMD **0.04 SD lower** (0.14 lower to 0.05 higher)	⊕◯◯◯ Very low	IMPORTANT
Cardoso E et al (2005) [16]												
**5**	randomised trials	very serious[a]	very serious[a]	not serious	not serious	publication bias strongly suspected[a]	122	123	-	SMD **0.95 SD lower** (1.17 lower to 0.74 lower)	⊕◯◯◯ Very low	CRITICAL
Rosales R et al (2008) [15]												
**9**	randomised trials	not serious[b]	very serious[c]	not serious	not serious	publication bias strongly suspected[a]	236	228	-	SMD **0.87 SD lower** (1.22 lower to 0.52 lower)	⊕◯◯◯ Very low	CRÍTICO
Elia AE et al (2009) [14]												
**11**	randomised trials	serious[d]	very serious[a]	not serious	not serious	publication bias strongly suspected[a]	89	90	-	SMD **0.98 SD lower** (1.17 lower to 0.78 lower)	⊕◯◯◯ Very low	IMPORTANTE
Koog YH and Min BI (2010) [41]												
**15**	randomised trials	serious[d]	very serious[c]	not serious	not serious	publication bias strongly suspected[a]	309	288	-	SMD **0.27 SD lower**	⊕◯◯◯ Very low	NOT IMPORTANT

										(0.8 lower to 0.26 higher)		
Baker Ja and Pereira G (2013) [13]												
**37**	randomised trials	serious[d]	very serious[c]	not serious	not serious	publication bias strongly suspected[a]	723	551	-	SMD **0.88 SD lower** (1.14 lower to 0.63 lower)	⊕◯◯◯ Very low	CRITICAL
Wu T et al (2016) [12]												
**7**	randomised trials	serious[d]	serious[e]	not serious	not serious	publication bias strongly suspected[f]	293	301	-	SMD **0.66 SD lower** (1.11 lower to 0.22 lower)	⊕◯◯◯ Very low	CRITICAL
Dong Y et al (2017) [11]												
**22**	randomised trials	serious[d]	serious[g]	not serious	not serious	publication bias strongly suspected[f]	902	902	-	SMD **0.81 SD lower** (0.93 lower to 0.68 lower)	⊕◯◯◯ Very low	CRITICAL
Guyot et al (2019) [18]												
**10**	randomised trials	serious[a]	very serious[c]	not serious	not serious	publication bias strongly suspected[a]	130	130	-	SMD **0.1 SD lower** (0.3 lower to 0.1 higher)	⊕◯◯◯ Very low	NOT IMPORTANT
Blumetti FC et al (2019) [19]												
**31**	randomised trials	very serious[h]	not serious[e]	not serious	not serious	publication bias strongly suspected[a]	33	32	-	SMD **0.42 SD higher** (0.65 lower to 0.18 lower)	⊕◯◯◯ Very low	IMPORTANT
Sun LC et al (2019) [10]												

**27**	randomised trials	very serious[h]	very serious[c]	not serious	not serious	none	234	324	-	SMD **0.76 SD lower** (0.97 lower to 0.55 lower)	⊕◯◯◯ Very low	CRITICAL
Jia S et al (2020) [42]												
**10**	randomised trials	very serious[h]	serious[g]	not serious	not serious	publication bias strongly suspected[a]	475	475	-	SMD **0.33 SD lower** (0.54 lower to 0.12 lower)	⊕◯◯◯ Very low	CRÍTICO
Doan TN et al (2021) [38]												
**12**	randomised trials	not serious[b]	serious[g]	not serious	not serious	publication bias strongly suspected[i]	524	504	-	SMD **0.45 SD lower** (0.73 lower to 0.18 lower)	⊕⊕◯◯ Low	CRITICAL
Ojardias E et al (2022) [17]												
**37**	randomised trials	serious[d]	very serious[a]	not serious	not serious	publication bias strongly suspected[a]	1505	1506	-	SMD **0.11 SD lower** (0.18 lower to 0.04 lower)	⊕◯◯◯ Very low	CRITICAL

CI: confidence interval; **SMD:** standardised mean difference

a. Non reported

b. Low risk of bias

c. Considerable heterogeneity

d. Some concerns

e. Moderate heterogeneity

f. Small number of studies

g. Substantial heterogeneity

h. High risk of bias

i. Egger's test statistically significant
